# Dose-Dependent Glutathione Modulation of Physiological Responses and Metal Accumulation in *Salix variegata* Under Cadmium Stress

**DOI:** 10.3390/plants15182801

**Published:** 2026-09-12

**Authors:** Renxiu Yao, Jian Li, Youwei Zuo, Nana Long, Hongping Deng

**Affiliations:** School of Life Sciences, Southwest University, Beibei, Chongqing 400715, China

**Keywords:** *Salix variegata*, glutathione, cadmium stress, dose-dependent response, tissue-specific response, redox homeostasis, cadmium accumulation, metal homeostasis

## Abstract

Exogenous glutathione (GSH) can alleviate cadmium (Cd) stress, yet physiological improvement and changes in internal Cd burden may not occur in parallel. *Salix variegata* seedlings were exposed for 10 d to 0 or 100 μM Cd with 0–1500 μM GSH. Across the full dose series, GSH responses were non-monotonic: 10 μM GSH increased root length by 67.3% under Cd stress, 250 μM GSH increased chlorophyll a, chlorophyll b, and total chlorophyll by 21.4%, 39.0%, and 25.7%, respectively, whereas 1500 μM GSH inhibited several growth and pigment traits. A common 2 × 2 subset (0/250 μM GSH × 0/100 μM Cd) was used to integrate root/leaf physiology with root/shoot element status. At 250 μM GSH, H_2_O_2_ and MDA decreased in both roots and leaves, while antioxidant and osmotic responses differed between tissues. Root Cd concentration increased by 64.1%, root Cd accumulation by 165%, and total Cd accumulation by 117%, despite a 24.1% decrease in shoot Cd concentration. Root Fe concentration and total Fe accumulation increased by 173.2% and 170%, respectively. These results show that GSH responses in *S. variegata* are dose- and tissue-dependent and that improved physiological status can coexist with greater Cd burden and altered essential-metal homeostasis.

## 1. Introduction

Cadmium (Cd) is a non-essential and highly phytotoxic metal that can be readily taken up by plant roots and interfere with multiple physiological and metabolic processes. Cd exposure commonly suppresses root development and biomass accumulation, disrupts photosynthetic pigment synthesis and photosynthetic performance, and induces oxidative injury through disturbances in cellular redox homeostasis [1,2,3]. Although Cd is not itself a redox-active metal, its accumulation can indirectly promote reactive oxygen species (ROS) production and disturb antioxidant metabolism, resulting in membrane lipid peroxidation and metabolic dysfunction [2,3]. Cd toxicity is also closely linked to mineral-nutrient homeostasis because Cd can compete with or interfere with the uptake, transport, and utilization of essential elements, including Fe, Zn, and Mn [1,4]. Consequently, plant responses to Cd involve not only control of oxidative damage but also coordinated adjustments in metal distribution and nutrient balance.

Woody species of *Salix* have attracted considerable interest for the phytoremediation of metal-contaminated environments because of their rapid growth, high biomass production, extensive root systems, and capacity to tolerate and accumulate heavy metals [5,6,7,8]. However, Cd accumulation and tolerance differ substantially among *Salix* species and genotypes, and high metal accumulation does not necessarily correspond to reduced physiological damage [5,6,7]. Recent physiological and multi-omics studies further indicate that willow responses to Cd involve extensive changes in root growth, antioxidant metabolism, and primary and secondary metabolic pathways [7]. *Salix variegata* Franch., a shrub willow occurring naturally in riparian habitats in southwestern China, has previously shown considerable Cd tolerance and accumulation capacity. Cd exposure inhibited biomass production and photosynthesis and altered antioxidant responses in this species, whereas exogenous organic acids partly alleviated these effects [9,10]. Importantly, organic-acid treatments that improved the physiological performance of Cd-exposed *S. variegata* could also increase total Cd accumulation [10], indicating that improved plant performance cannot necessarily be inferred from a reduction in internal Cd burden.

Glutathione (GSH) is a major low-molecular-weight thiol involved in plant redox regulation and metal-stress responses. It participates directly and indirectly in ROS scavenging, contributes to maintenance of cellular redox homeostasis, and provides the precursor for phytochelatin biosynthesis and subsequent metal chelation and sequestration [1,3,11]. Accordingly, exogenous GSH has been investigated as a means of modifying plant responses to Cd. GSH application has improved growth, chlorophyll status, and antioxidant responses in Cd-exposed wheat, oilseed rape, potato, purple flowering stalk, and *Pogostemon cablin* [12,13,14,15,16]. Nevertheless, the response to exogenous GSH is not uniform across species or physiological processes. In wheat, GSH increased root Cd concentration while decreasing Cd movement to the shoot [13], whereas foliar GSH application to *Brassica napus* alleviated Cd-induced oxidative stress despite increasing Cd concentrations and accumulation in both roots and shoots [12]. Recent transcriptomic and metabolomic studies have likewise shown that GSH-mediated responses to Cd involve multiple metabolic and regulatory processes rather than a single antioxidant pathway [14,15,16]. These observations indicate that GSH-mediated physiological protection and changes in Cd uptake or accumulation should be evaluated separately.

Another important consideration is that the effects of exogenous GSH may depend on application concentration. Several recent studies have demonstrated beneficial effects of selected GSH treatments under Cd stress [12,13,14,15,16], but comparatively less attention has been given to responses across a broad GSH concentration range. This distinction is important because redox-active compounds can produce different physiological outcomes depending on concentration and stress background, and responses of growth, photosynthetic pigments, ROS-related variables, and antioxidant enzymes may not reach their strongest effects at the same GSH concentration. A broad dose–response assessment is therefore needed to distinguish generally beneficial responses from concentration-specific effects and to identify conditions suitable for more integrated physiological analysis.

Cd-induced disruption of essential-metal homeostasis provides an additional dimension for evaluating GSH responses. Fe, Zn, and Mn contribute to photosynthesis, redox enzymes, electron transport, and numerous other metabolic processes, and their uptake and tissue distribution can be altered during Cd exposure [4,17]. Experimental manipulation of Fe availability has also been shown to modify Cd toxicity, oxidative status, and the concentrations of other mineral elements [17]. However, studies of exogenous GSH under Cd stress have generally focused more strongly on antioxidant responses and Cd concentrations than on the concurrent behavior of essential metals. Moreover, tissue metal concentration alone does not describe the actual amount of an element retained by a plant because concentration can change independently of biomass. Evaluation of concentration together with biomass-matched element accumulation is therefore useful for distinguishing changes in tissue concentration from changes in actual internal element burden. Recent approaches to limiting crop Cd accumulation include managing soil microorganisms and organic matter, applying nutrient amendments, and using boron to reinforce apoplastic barriers and regulate transmembrane transport [18,19]. These approaches underscore that Cd accumulation is governed jointly by rhizosphere chemistry and plant transport processes. Whether exogenous GSH can simultaneously reshape the physiological condition and actual Cd burden across a broad dose range remains insufficiently resolved in woody accumulators.

In the present study, *S. variegata* seedlings were exposed to 0 or 100 μM Cd together with a broad series of GSH concentrations (0, 10, 50, 250, 750, and 1500 μM). The experiment was evaluated at two complementary levels. The complete GSH series was used to characterize growth, root morphology, and photosynthetic pigment responses, whereas a common 2 × 2 subset comprising 0 or 250 μM GSH under 0 or 100 μM Cd was used to integrate root and leaf physiology with root and shoot element status. Additional GSH + Cd physiological treatments were retained to provide dose–response context. The objectives were to (i) determine whether growth and photosynthetic responses to GSH under Cd stress were concentration- and Cd-background-dependent; (ii) characterize root and leaf physiological responses associated with 250 μM GSH; (iii) determine whether changes in physiological performance were necessarily accompanied by a reduction in internal Cd burden; and (iv) assess the accompanying changes in Fe, Zn, and Mn homeostasis. Rather than defining a single universally optimal GSH concentration, this framework was used to resolve dose dependence and compare physiological condition with actual element burden across a common factorial subset.

## 2. Results

### 2.1. Growth and Morphological Responses Varied with Cd and GSH Treatments

**Figure 1:**
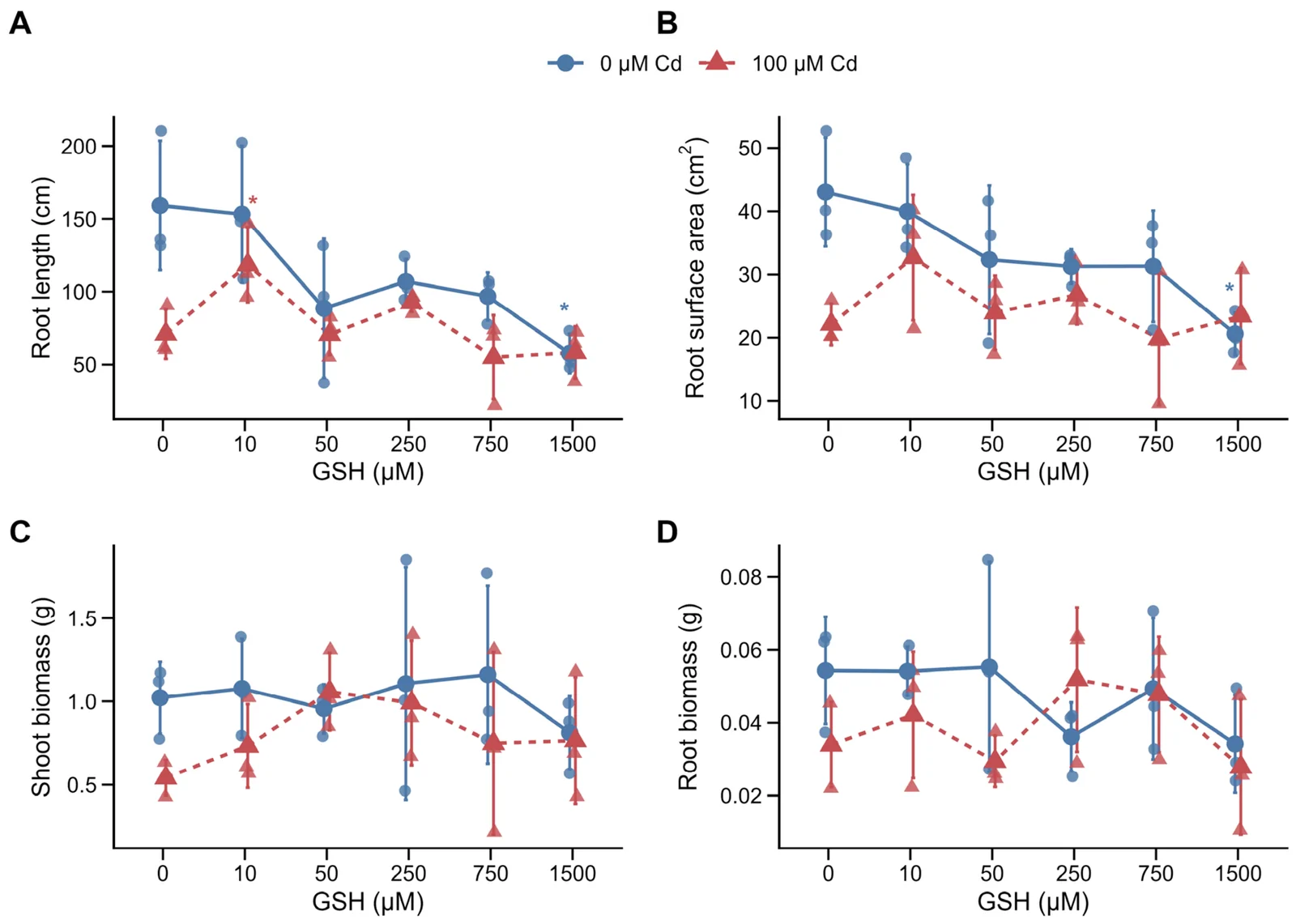
Growth responses of *Salix variegata* across the complete GSH dose series under control and Cd-stress conditions. Root length (**A**), root surface area (**B**), shoot biomass (**C**), and root biomass (**D**) were measured in plants supplied with 0, 10, 50, 250, 750, or 1500 μM glutathione (GSH) in the absence or presence of 100 μM Cd. Small symbols represent individual biological replicates, and large symbols with error bars represent means ± SD (*n* = 3). Blue circles and solid lines indicate 0 μM Cd, whereas red triangles and dashed lines indicate 100 μM Cd. Blue asterisks indicate significant Dunnett-adjusted comparisons with CK within the Cd-free background, and red asterisks indicate significant comparisons with the Cd-only treatment within the 100 μM Cd background. * adjusted *p* < 0.05.

Cd and GSH affected individual growth and morphological traits, but no significant Cd × GSH interaction was detected for any of the nine measured traits (all interaction *p* > 0.13; Figure 1 and Figure S1, and Table S2). Significant Cd main effects were detected for root length (*p* = 0.0019), root surface area (*p* = 0.0034), leaf length (*p* = 0.0299), and leaf width (*p* = 0.0047). GSH concentration significantly affected root length (*p* < 0.001), root surface area (*p* = 0.0495), and leaf width (*p* = 0.0300).

In the absence of GSH, mean root length decreased from 159.24 cm in CK to 70.83 cm under Cd treatment, whereas mean root surface area decreased from 43.03 to 22.12 cm^2^. Within the Cd-stressed treatments, 10 μM GSH produced the only significant increase in root length relative to Cd alone, increasing the mean from 70.83 to 118.53 cm (+67.3%; Dunnett-adjusted *p* = 0.0455; Figure 1A and Table S3). Root length at 250 μM GSH was 30.7% higher than that in Cd alone, but this difference was not significant (adjusted *p* = 0.552).

Significant reductions were observed at the upper end of the GSH concentration range. In Cd-free plants, 1500 μM GSH reduced root length by approximately 63.9% and root surface area by approximately 52.1% relative to CK (adjusted *p* < 0.05). Leaf area was reduced by 31.4% at 750 μM GSH compared with CK (adjusted *p* = 0.042). Under Cd stress, 1500 μM GSH significantly reduced leaf width by approximately 18.3% relative to Cd alone (adjusted *p* < 0.05; Figure S1 and Table S3). No significant Dunnett-adjusted differences in shoot or root biomass were detected among the GSH treatments within the Cd-stressed background.

### 2.2. Photosynthetic Pigments Showed Strong Cd × GSH Interactions

**Figure 2:**
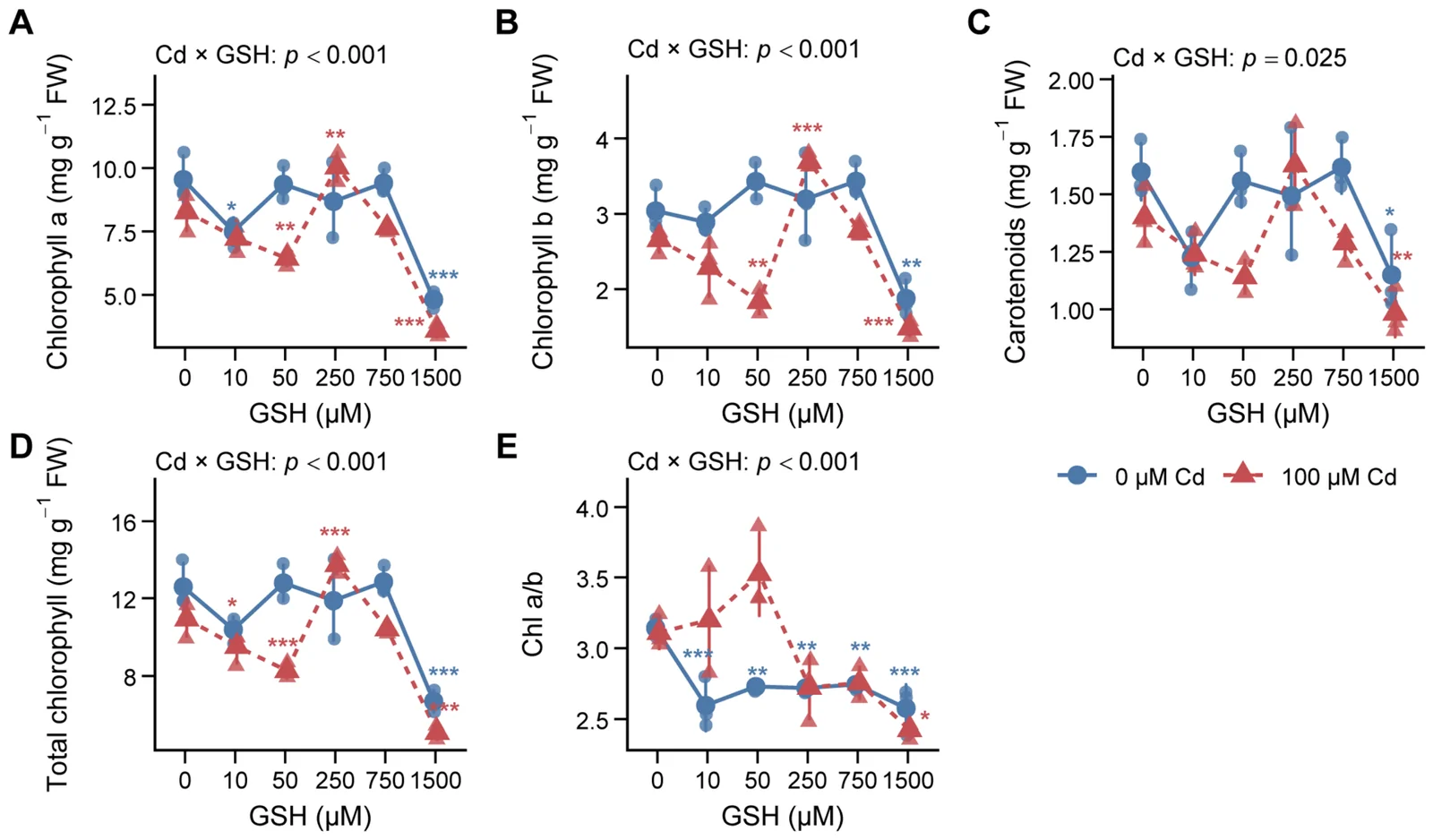
Non-monotonic effects of exogenous GSH on photosynthetic pigments of *Salix variegata* under Cd stress. Chlorophyll a (**A**), chlorophyll b (**B**), carotenoids (**C**), total chlorophyll (**D**), and the chlorophyll a/b ratio (**E**) were determined in plants supplied with 0, 10, 50, 250, 750, or 1500 μM GSH under 0 or 100 μM Cd. Chlorophyll and carotenoid contents are expressed as mg g^−1^ FW; Chl a/b is dimensionless. Small symbols represent individual biological replicates, and large symbols with error bars represent means ± SD (*n* = 3). The *p* value shown in each panel is the Cd × GSH interaction from the Type III factorial ANOVA. Blue asterisks indicate Dunnett-adjusted comparisons with CK within the Cd-free background, whereas red asterisks indicate comparisons with the Cd-only treatment within the Cd background. * *p*_adj < 0.05, ** *p*_adj < 0.01, and *** *p*_adj < 0.001.

All five photosynthetic pigment variables showed significant Cd × GSH interactions, including Chl a (*p* = 0.00042), Chl b (*p* < 0.001), Car (*p* = 0.0250), Chl t (*p* = 0.00018), and Chl a/b (*p* = 0.00024; Figure 2 and Table S4). Significant GSH main effects were also detected for all five pigment variables.

The responses under Cd stress differed markedly among GSH concentrations. At 50 μM GSH, Chl a decreased by 22.1% relative to Cd alone (Dunnett-adjusted *p* = 0.0018), Chl b decreased by 31.0% (adjusted *p* = 0.0010), and Chl t decreased by 24.3% (adjusted *p* < 0.001). The 18.6% reduction in Car at this concentration did not reach statistical significance (adjusted *p* = 0.058).

In contrast, 250 μM GSH significantly increased several chlorophyll variables under Cd stress. Compared with Cd alone, G3Cd showed a 21.4% increase in Chl a (adjusted *p* = 0.0024), a 39.0% increase in Chl b (adjusted *p* < 0.001), and a 25.7% increase in Chl t (adjusted *p* < 0.001). Car increased by 16.1%, but this difference was not significant (adjusted *p* = 0.112), and the Chl a/b ratio also did not differ significantly between G3Cd and Cd.

At 1500 μM GSH, all measured pigment variables were reduced under Cd stress. Relative to Cd alone, Chl a, Chl b, Car, Chl t, and Chl a/b decreased by 56.5%, 44.1%, 29.9%, 53.5%, and 22.0%, respectively (all adjusted *p* < 0.05; Figure 2 and Table S5). In the absence of Cd, 1500 μM GSH also significantly reduced Chl a, Chl b, Car, and Chl t relative to CK (adjusted *p* < 0.05).

### 2.3. The 250 μM GSH Treatment Altered Root and Leaf Physiological Profiles

**Figure 3:**
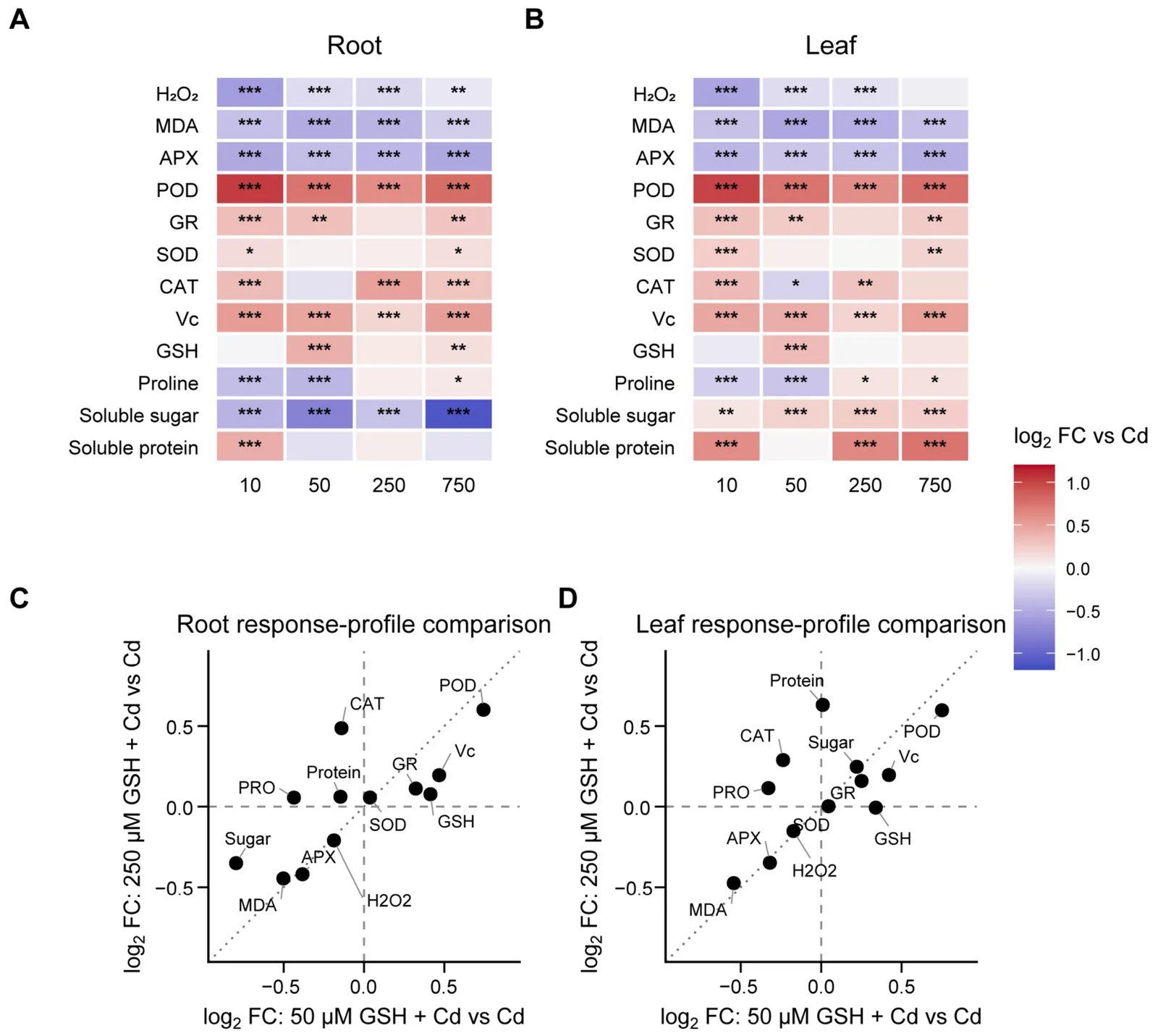
Physiological profiles and Cd × GSH interaction effects in the 250 μM GSH-centered 2 × 2 design. The core groups were CK, G3, Cd, and G3Cd, corresponding to Cd at 0 or 100 μM and GSH at 0 or 250 μM. Heatmaps show standardized physiological profiles in roots (**A**) and leaves (**B**) for H_2_O_2_, MDA, APX, POD, GR, SOD, CAT, Vc, GSH, proline, soluble sugar, and soluble protein. Values are mean z-scores calculated from biological-replicate data after standardization within each tissue and variable across the four core groups. Asterisks in G3 and G3Cd cells indicate planned GSH simple effects (G3 vs. CK and G3Cd vs. Cd, respectively) with Holm-adjusted *p* values. Panels (**C**,**D**) show partial ηp^2^ for the Cd × GSH interaction in roots and leaves, respectively; significance labels beside the points correspond to the interaction *p* values from the Type III ANOVA. ns, not significant; * *p* < 0.05, ** *p* < 0.01, and *** *p* < 0.001.

The CK/G3/Cd/G3Cd 2 × 2 analysis revealed significant Cd × GSH interactions for 9 of the 12 physiological variables in roots and 10 of the 12 variables in leaves (Figure 3 and Table S6). In roots, significant interactions were detected for H_2_O_2_, MDA, APX, POD, GR, CAT, GSH, soluble sugar, and soluble protein, whereas the interactions for SOD, Vc, and proline were not significant. In leaves, significant interactions were detected for H_2_O_2_, MDA, APX, POD, GR, CAT, Vc, GSH, soluble sugar, and soluble protein, whereas SOD and proline showed no significant interaction (Figure 3C,D).

In roots under Cd-free conditions, G3 showed significantly higher APX activity and higher Vc, GSH, H_2_O_2_, proline, MDA, soluble sugar, and soluble protein contents than CK (Holm-adjusted *p* < 0.05; Figure S2 and Table S7). In contrast, POD, GR, and CAT activities were significantly lower in G3 than in CK. Root SOD activity did not differ significantly between these treatments.

Under Cd stress, the G3Cd root response differed among variables. Compared with Cd alone, G3Cd had significantly lower APX activity and lower H_2_O_2_, MDA, and soluble sugar contents, whereas POD and CAT activities and Vc content were significantly higher (Holm-adjusted *p* < 0.05). Root GR activity, GSH content, proline content, SOD activity, and soluble protein content did not differ significantly between Cd and G3Cd (Figure S2 and Table S7).

In leaves under Cd-free conditions, APX activity and Vc, GSH, H_2_O_2_, proline, MDA, and soluble protein contents were significantly higher in G3 than in CK, whereas POD, GR, and CAT activities and soluble sugar content were significantly lower (Holm-adjusted *p* < 0.05). Leaf SOD activity did not differ significantly.

Under Cd stress, G3Cd showed significantly lower APX activity, H_2_O_2_ content, and MDA content than Cd. In contrast, POD, GR, and CAT activities and Vc, proline, soluble sugar, and soluble protein contents were significantly higher in G3Cd (Holm-adjusted *p* < 0.05). Leaf GSH content and SOD activity did not differ significantly between G3Cd and Cd (Figure S3 and Table S7).

The supporting dose comparisons under 100 μM Cd showed that physiological responses were not restricted to 250 μM GSH. Significant differences from Cd alone occurred at one or more of the 10, 50, 250, and 750 μM GSH treatments for most physiological variables, and the direction and magnitude of these changes varied among doses and between roots and leaves (Figure S4 and Table S8).

### 2.4. The 250 μM GSH Treatment Altered Tissue Cd Concentrations and Increased Root and Total Cd Accumulation

**Figure 4:**
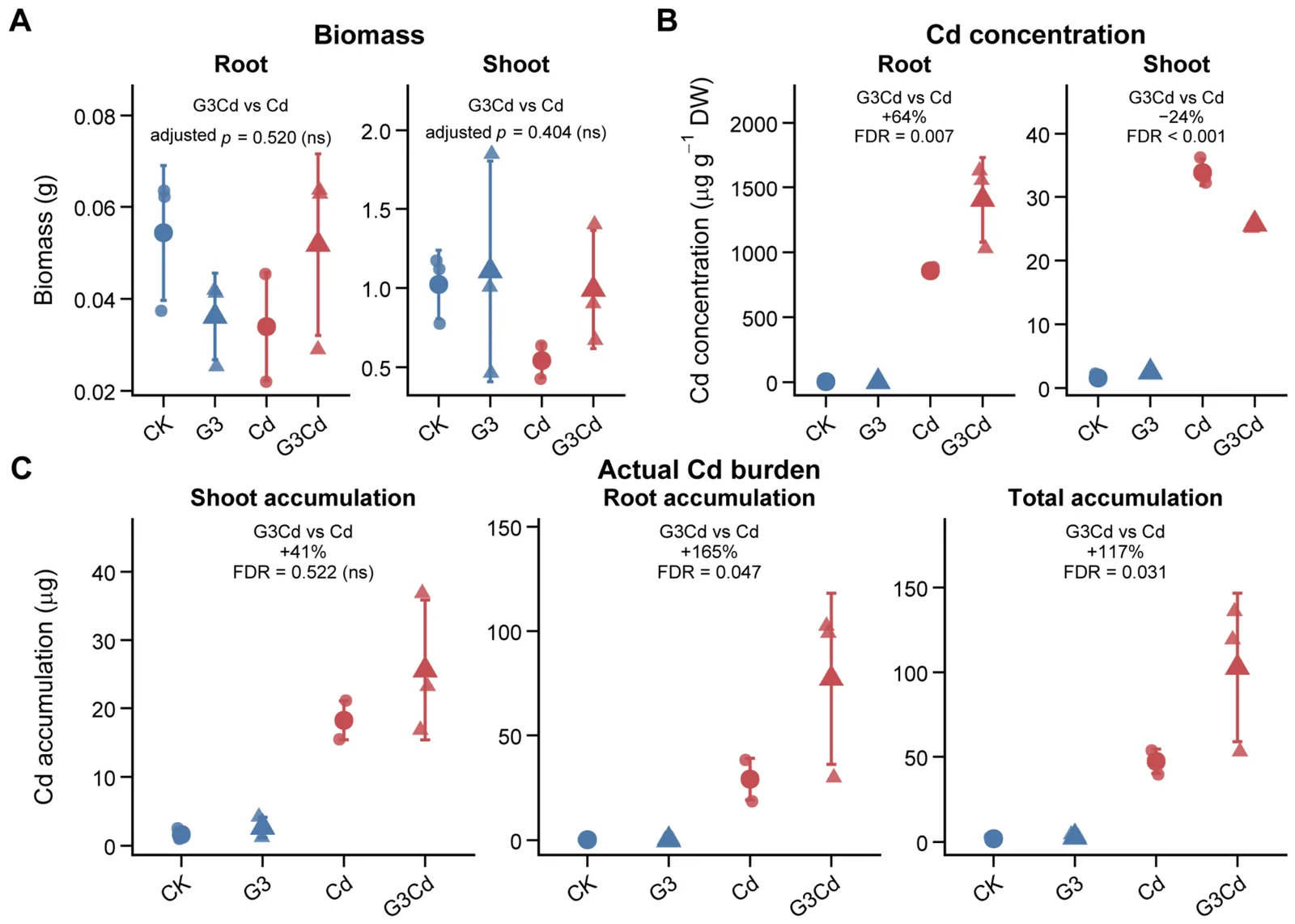
Biomass, tissue Cd concentration, and actual Cd burden in the 250 μM GSH-centered treatment set. Root and shoot biomass (**A**), root and shoot Cd concentrations (**B**), and shoot, root, and total Cd accumulation (**C**) are shown for CK, G3, Cd, and G3Cd plants. G3 denotes 250 μM GSH without Cd, and G3Cd denotes 250 μM GSH supplied with 100 μM Cd. Cd concentrations are expressed as μg g^−1^ DW. Cd accumulation was calculated by multiplying tissue Cd concentration by the corresponding tissue biomass from the same biological replicate and is expressed in μg. Small symbols represent individual biological replicates, and large symbols with error bars represent means ± SD (*n* = 3). Blue and red symbols indicate treatments without Cd and with 100 μM Cd, respectively; circles and triangles indicate 0 and 250 μM GSH, respectively. Panel (**A**) reports the Dunnett-adjusted comparison between G3Cd and Cd. Panels (**B**,**C**) report the percentage change and BH-FDR-adjusted *p* value for G3Cd versus Cd. ns, not significant.

Biomass, tissue Cd concentration, and Cd accumulation were examined together for CK, G3, Cd, and G3Cd (Figure 4). Mean shoot biomass increased from 0.542 g in Cd to 0.991 g in G3Cd (+83.0%), whereas mean root biomass increased from 0.0340 to 0.0518 g (+52.6%). Neither G3Cd-versus-Cd biomass comparison was significant after Dunnett adjustment (adjusted *p* > 0.05; Figure 4A).

Tissue Cd concentrations responded differently in roots and shoots. Root Cd concentration was 64.1% higher in G3Cd than in Cd (FDR = 0.007), whereas shoot Cd concentration was 24.1% lower in G3Cd (FDR < 0.001; Figure 4B and  Figure S5, and Table S10).

When tissue concentrations were combined with the corresponding biomass from the same biological replicate, shoot Cd accumulation increased from 18.23 to 25.62 μg (+40.6%), but this difference was not significant (FDR = 0.522). Root Cd accumulation increased from 29.11 to 77.14 μg (+165.0%; FDR = 0.047), and total Cd accumulation increased from 47.34 to 102.77 μg (+117.1%; FDR = 0.031; Figure 4C and  Figure S6, and Table S11).

The mean proportion of total Cd present in the root fraction increased from approximately 60.2% in Cd to 71.7% in G3Cd. This difference in root Cd allocation was not statistically significant after multiple-testing correction (Table S11).

### 2.5. The 250 μM GSH Treatment Altered Fe, Zn, and Mn Concentrations and Fe Accumulation

**Figure 5:**
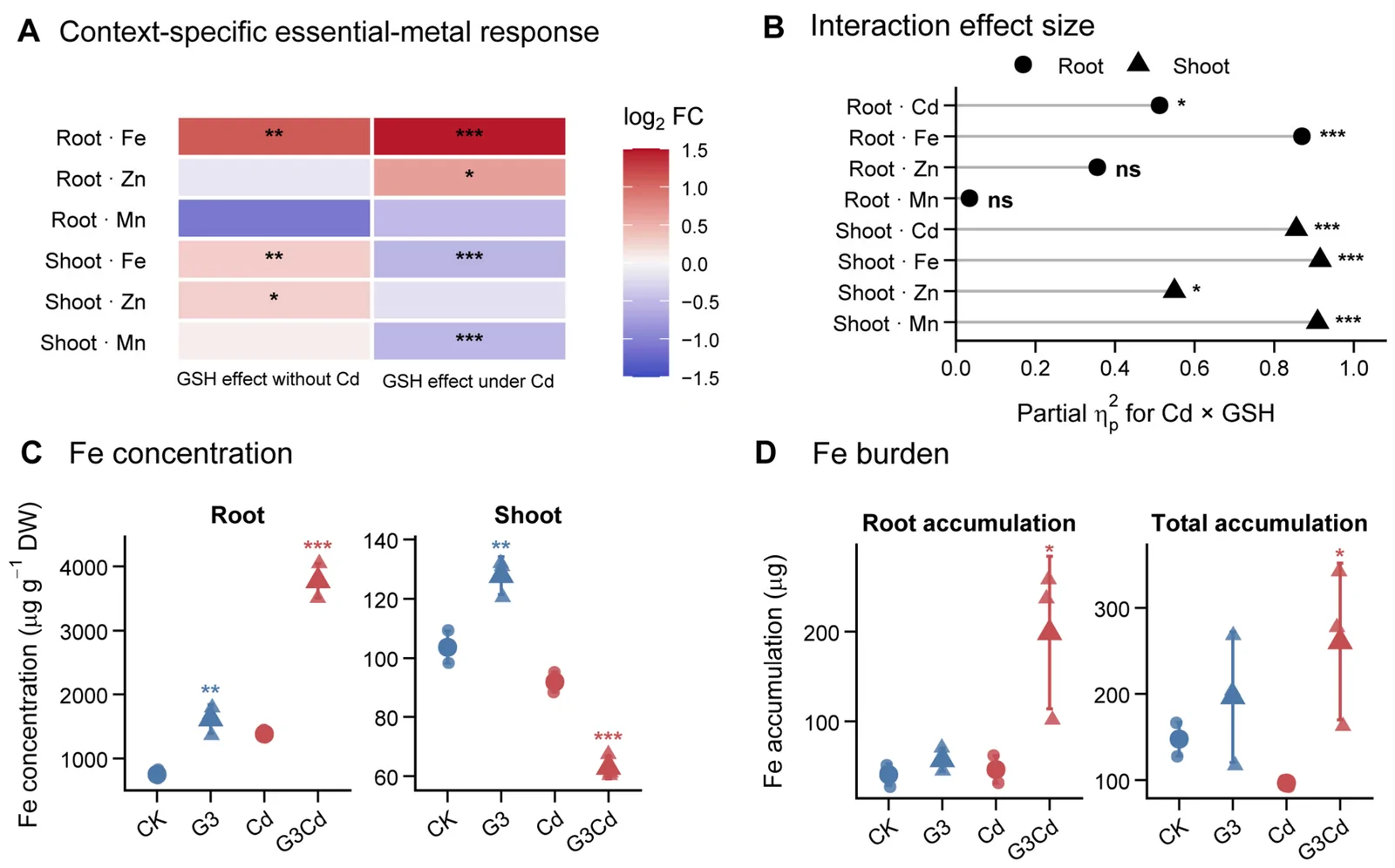
Context-dependent essential-metal homeostasis responses associated with 250 μM GSH. (**A**) Log_2_ fold changes in Fe, Zn, and Mn concentrations following GSH treatment in the absence of Cd (G3 vs. CK) or under Cd stress (G3Cd vs. Cd), shown separately for roots and shoots. Asterisks indicate BH-FDR-adjusted simple-effect comparisons. (**B**) Partial η_p_^2^ values for the Cd × GSH interaction for Cd, Fe, Zn, and Mn concentrations in roots and shoots; significance labels indicate the interaction *p* values from the Type III ANOVA. In panel (**B**), circles and triangles represent roots and shoots, respectively. (**C**) Fe concentrations in roots and shoots of CK, G3, Cd, and G3Cd plants. In panels (**C**,**D**), blue and red data symbols indicate treatments without Cd and with 100 μM Cd, respectively; circles and triangles indicate 0 and 250 μM GSH, respectively. In panel (**C**), blue asterisks indicate G3 versus CK, whereas red asterisks indicate G3Cd versus Cd, based on BH-FDR-adjusted comparisons. (**D**) Root and total Fe accumulation, with red asterisks indicating BH-FDR-adjusted comparisons between G3Cd and Cd.Small symbols represent individual biological replicates, and large symbols with error bars represent means ± SD (*n* = 3). ns, not significant; * *p* or FDR < 0.05, ** *p* or FDR < 0.01, and *** *p* or FDR < 0.001, as appropriate for the statistical test displayed.

The concentrations of Fe, Zn, and Mn differed between GSH treatments and between Cd backgrounds (Figure 5 and Figure S5, and Table S9 and Table S10). In the absence of Cd, G3 had significantly higher Fe concentrations in both roots and shoots than CK (FDR < 0.05). Shoot Zn concentration also differed significantly between G3 and CK, whereas root Zn and root and shoot Mn concentrations did not show significant GSH effects.

Under Cd stress, the largest concentration changes were observed for Fe. Root Fe concentration was 173.2% higher in G3Cd than in Cd (FDR = 8.61 × 10^−7^), whereas shoot Fe concentration was 31.4% lower (FDR = 1.27 × 10^−4^). Root Zn concentration increased by 54.5% in G3Cd (FDR = 0.049), whereas the 11.8% decrease in shoot Zn concentration was not significant. Root Mn concentration decreased by 29.4%, but the difference was not significant, whereas shoot Mn concentration decreased by 30.8% (FDR = 1.72 × 10^−5^; Figure 5A and Figure S5, and Table S10). Significant Cd × GSH interactions were detected for several element–tissue combinations, with the corresponding partial ηp^2^ values shown in Figure 5B and Table S9.

Element accumulation showed the strongest significant G3Cd-versus-Cd changes for Fe. Root Fe accumulation increased from 46.60 to 198.66 μg, corresponding to an increase of 326.3% (FDR = 0.011). Total Fe accumulation increased from 96.56 to 260.76 μg (+170.0%; FDR = 0.032), whereas shoot Fe accumulation increased by 24.3% but was not significant (FDR = 0.747; Figure 5D and Figure S6, and Table S11).

For Zn, shoot, root, and total accumulation increased by 61.9%, 139.5%, and 64.3%, respectively, but none of these differences remained significant after FDR correction. Shoot, root, and total Mn accumulation changed by 28.0%, 9.3%, and 26.9%, respectively, and none of these comparisons was significant (Figure S6 and Table S11).

## 3. Discussion

### 3.1. GSH Responses to Cd Stress Were Concentration- and Trait-Dependent

The present study showed that the effects of exogenous GSH on *S. variegata* could not be described by a simple concentration-dependent improvement in plant performance. Growth and morphological traits showed no significant Cd × GSH interactions across the complete GSH concentration series, and only 10 μM GSH significantly increased root length relative to the Cd-only treatment. In contrast, all five photosynthetic pigment variables showed significant Cd × GSH interactions, with markedly different responses among GSH concentrations. In particular, 50 μM GSH further reduced several chlorophyll variables under Cd stress, whereas 250 μM GSH increased Chl a, Chl b, and total chlorophyll. At 1500 μM GSH, substantial reductions in growth-related traits and photosynthetic pigments occurred in both Cd-free and Cd-treated plants. These findings indicate that exogenous GSH was not physiologically neutral across the tested concentration range and that increasing GSH supply did not result in progressively stronger protection.

Most previous studies evaluating exogenous GSH under Cd stress have focused on one or a limited number of GSH concentrations and have generally reported improvements in growth, photosynthesis, or antioxidant performance [12,13,14,15,16,20,21]. For example, GSH application improved photosynthetic performance and antioxidant responses in *Brassica napus*, potato, purple flowering stalk, and *Pogostemon cablin* under Cd stress [12,14,15,16,21]. However, GSH is not only an antioxidant substrate but also participates in redox signalling, sulfur metabolism, thiol homeostasis, protein modification, and the synthesis of metal-binding phytochelatins [20]. Consequently, altering the external GSH supply may affect multiple processes simultaneously rather than simply increasing antioxidant capacity. The growth inhibition and pigment loss observed at the highest GSH concentration in the present study therefore emphasize the importance of considering GSH concentration as an experimental factor rather than assuming that higher supplementation is necessarily more effective.

A further distinction was evident among response types. The strongest significant growth response under Cd stress occurred at 10 μM GSH, whereas the clearest preservation of chlorophyll occurred at 250 μM. Similarly, the supporting physiological dose analysis showed that 50 and 250 μM GSH did not produce identical responses across redox-related variables. Thus, no single GSH concentration was uniformly optimal for all measured traits. This trait dependence is important when interpreting exogenous-GSH experiments because growth, photosynthetic pigments, antioxidant variables, and metal accumulation represent different levels of plant response. Accordingly, the 250 μM treatment within the common factorial subset should be interpreted as one physiologically informative concentration for cross-endpoint integration rather than as a universally optimal GSH concentration.

### 3.2. The 250 μM GSH Treatment Reconfigured Rather than Uniformly Enhanced Physiological Responses

The CK/G3/Cd/G3Cd factorial analysis showed that GSH strongly modified the physiological response to Cd. Significant Cd × GSH interactions occurred for most measured variables in both roots and leaves, demonstrating that the effect of 250 μM GSH depended on the Cd background. Under Cd stress, G3Cd generally showed lower H_2_O_2_ and MDA contents than Cd alone in both organs. These changes provide direct evidence that the oxidative state of the tissues differed between Cd and G3Cd. Similar decreases in ROS accumulation and membrane lipid peroxidation following GSH application have been reported in Cd-exposed *B. napus*, purple flowering stalk, and *P. cablin* [12,15,16,21].

Nevertheless, the antioxidant-enzyme responses in *S. variegata* were not uniformly increased by GSH. Under Cd stress, POD and CAT activities increased in several comparisons, whereas APX activity decreased, SOD remained largely unchanged, and GR responses differed between roots and leaves. Therefore, the physiological response is more appropriately described as a reconfiguration of the antioxidant system than as a general activation of all antioxidant enzymes. This interpretation also agrees with the multifunctional role of glutathione in plant cells. GSH interacts with the ascorbate–glutathione cycle, thiol redox regulation, sulfur metabolism, metal chelation, signalling pathways, and protein glutathionylation, and changes in one component of this network do not necessarily require parallel changes in every antioxidant enzyme [20]. Proteomic analyses of Cd-stressed *B. napus* similarly showed that GSH-responsive proteins extend beyond classical ROS-scavenging enzymes to photosynthesis, sulfur assimilation, energy metabolism, and metal detoxification pathways [21].

Although several GSH-associated responses were shared by roots and leaves, the two tissues were not fully concordant. Under Cd stress, G3Cd showed lower H_2_O_2_ and MDA contents and lower APX activity than Cd alone in both tissues, whereas SOD activity and bulk GSH content were not significantly altered. In contrast, GR activity, proline, and soluble protein increased significantly in leaves but not in roots, while soluble sugar decreased in roots and increased in leaves. This combination of shared oxidative responses and divergent antioxidant and osmotic responses indicates tissue-dependent physiological adjustment rather than a uniform whole-plant response. Comparable organ specificity has been reported in kenaf and willow under Cd stress [22,23,24]. Roots represent the primary site of Cd exposure and accumulation, whereas leaves must maintain photosynthetic and metabolic functions under the downstream effects of Cd stress. However, because the elemental shoot fraction in the present study comprised pooled stems and leaves, these root-leaf physiological contrasts should not be directly equated with root-shoot elemental differences.

An additional feature of the present dataset was that exogenous GSH itself markedly changed several physiological variables in Cd-free plants. G3 differed from CK in APX, POD, GR, CAT, Vc, GSH, H_2_O_2_, MDA, osmotic-related variables, and other endpoints, depending on tissue. This observation reinforces that 250 μM GSH represented an active physiological treatment rather than an inert supplement whose effects emerged only after Cd exposure. Moreover, although G3Cd displayed lower H_2_O_2_ and MDA contents than Cd, the measured bulk GSH content was not significantly higher in G3Cd than in Cd in either tissue. GSH concentration measured at a single harvest point does not directly represent GSH turnover, the GSH/GSSG redox state, or flux through GSH-dependent pathways [20]. Consequently, the present data support an association between exogenous GSH and an altered redox state but do not demonstrate that this response was driven simply by accumulation of a larger endogenous GSH pool.

The physiological dose-context analysis further showed that several conventional redox variables responded strongly at 50 μM GSH, whereas pigment preservation was most evident at 250 μM. Therefore, the degree of recovery in individual antioxidant or oxidative-stress indicators did not directly predict the chlorophyll response across GSH concentrations. Recent transcriptomic, proteomic, and metabolomic studies likewise indicate that GSH-mediated Cd tolerance involves processes extending beyond ROS scavenging, including phenylpropanoid metabolism, hormone signalling, sulfur assimilation, lipid metabolism, and photosynthetic regulation [14,15,16,21]. The current results are consistent with this broader view, although the present physiological dataset alone cannot identify the molecular pathways responsible for the different responses of 50 and 250 μM GSH.

### 3.3. Improved Physiological Performance Was Not Associated with a Lower Whole-Plant Cd Burden

A central result of this study was the clear separation between tissue Cd concentration and actual Cd accumulation. Compared with Cd alone, G3Cd had a 64.1% higher root Cd concentration but a 24.1% lower shoot Cd concentration. Considering concentration alone could therefore suggest that 250 μM GSH reduced the amount of Cd reaching aboveground tissues. However, this interpretation was not supported when tissue biomass was incorporated. Shoot Cd accumulation did not decrease significantly, whereas root Cd accumulation increased by 165% and total plant Cd accumulation increased by 117%. Thus, the improved pigment and physiological status of G3Cd occurred despite a substantially greater internal Cd burden.

The distinction between concentration and accumulation is particularly important when treatments simultaneously affect plant biomass. In the present study, mean shoot biomass was higher in G3Cd than in Cd, although this difference was not statistically significant. Consequently, the lower shoot Cd concentration did not correspond to a lower absolute amount of Cd in the shoot. These results demonstrate why a decrease in tissue concentration should not automatically be interpreted as reduced uptake or reduced translocation when biomass also changes. Because root-to-shoot Cd flux was not directly measured, the current results do not support the stronger conclusion that GSH restricted Cd translocation.

The coexistence of greater Cd accumulation and reduced physiological injury is not unprecedented in woody plants. Ding et al. [25] showed that exogenous GSH increased Cd^2+^ influx and total Cd accumulation in *Populus* × *canescens* while reducing Cd-induced H_2_O_2_ accumulation and improving Cd tolerance. Their molecular analyses further indicated increased expression of genes associated with Cd uptake, chelation, and vacuolar sequestration [22]. Although those molecular mechanisms cannot be assumed for *S. variegata*, the physiological pattern is relevant because it demonstrates that greater Cd accumulation and improved stress performance can occur simultaneously in a woody species.

A particularly relevant comparison is available within *S. variegata* itself. Zhang et al. [10] found that citric, tartaric, and malic acids reduced oxidative injury and improved physiological performance under Cd stress while increasing total plant Cd accumulation to approximately 178–209% of that in the Cd-only treatment. The current GSH results therefore extend an existing characteristic observed in this species: improved physiological performance under Cd exposure does not necessarily require a reduction in whole-plant Cd accumulation. This pattern may be particularly relevant to Cd-tolerant woody plants, in which tolerance can depend on the ability to accommodate or detoxify internal Cd rather than simply excluding it. Recent work in *Salix caprea* provides a related example: under Cd exposure, sufficient phosphorus increased root Cd accumulation while reducing root H_2_O_2_ and oxidative stress [26]. Together, these willow studies show that improved physiological status can coexist with a greater root Cd burden under stress-modifying treatments.

GSH and GSH-derived phytochelatins provide several potential biochemical routes for Cd detoxification because thiol groups can bind Cd and facilitate intracellular sequestration [1,3,20,27]. Experimental evidence in Marchantia polymorpha has demonstrated coordinated roles of GSH and phytochelatins in intracellular and extracellular Cd detoxification [27]. In willow, Cd tolerance has also been associated with AsA–GSH metabolism and tissue/subcellular Cd handling [24], while lignin-rich root cell walls can retain a large proportion of Cd [28]. However, the present experiment did not determine phytochelatins, Cd chemical forms, subcellular Cd distribution, cell-wall binding, or transporter expression. It would therefore be premature to attribute the elevated root Cd burden of G3Cd to enhanced chelation, vacuolar sequestration, or a specific transporter pathway. The data demonstrate tolerance in the presence of a larger internal Cd burden, whereas the cellular mechanism allowing that condition remains to be resolved.

From a phytoremediation perspective, the combination of maintained physiological performance and increased total Cd accumulation is potentially useful for a woody species. Recent work with willow genotype NJU513 similarly demonstrated that treatment-induced increases in plant biomass can enhance the total amount of Cd extracted even when concentration responses alone do not fully describe remediation performance [6]. However, the current experiment was conducted hydroponically for 10 d, and most of the additional Cd burden occurred in roots. Therefore, field-scale phytoextraction efficiency should not be inferred directly from these results.

### 3.4. The 250 μM GSH Treatment Was Associated with Selective Remodeling of Essential-Metal Homeostasis

The targeted element analysis showed that the response associated with 250 μM GSH was not limited to Cd. Fe displayed the strongest response: under Cd stress, G3Cd increased root Fe concentration by 173.2% while decreasing shoot Fe concentration by 31.4%. When biomass was considered, root Fe accumulation increased by 326.3% and total Fe accumulation increased by 170%. Zn and Mn showed different response patterns; root Zn concentration increased significantly, whereas shoot Zn did not change significantly, and shoot Mn concentration decreased significantly without a corresponding significant change in total Mn accumulation. These element-specific and tissue-specific patterns indicate selective remodeling of essential-metal homeostasis rather than a uniform increase or decrease in mineral uptake.

The root–shoot contrast was particularly evident for Fe and Cd, which changed in opposite directions at the concentration level after 250 μM GSH application, while Zn and Mn displayed different organ-specific patterns. Cd-induced disruption of mineral nutrition can likewise vary among plant tissues; a recent physiological and multi-omics study in Solanum nigrum reported tissue-dependent changes in mineral-element profiles together with distinct physiological responses in roots and leaves [29]. In the present study, these root–shoot elemental contrasts are therefore most appropriately interpreted as evidence of tissue-dependent metal-homeostasis remodeling rather than as direct proxies for the root–leaf biochemical responses. A recent study in Salix caprea further showed that phosphorus supply under Cd stress jointly altered root antioxidant status, Cd handling, and mineral uptake, including Mn [30], illustrating that nutrient and redox responses can be coupled within a tissue-specific stress response.

Interactions between Cd and essential divalent metals are expected because Cd can enter plants through transport systems that normally function in Fe, Zn, or Mn acquisition and distribution [4]. Recent work has further demonstrated that the direction of these interactions is highly dependent on nutrient status, transporter specificity, and plant species. Fe supplementation can reduce Cd uptake in wheat through combined effects of rhizosphere chemistry, competitive absorption, and physiological regulation [31], whereas Mn can compete with Cd for uptake through Mn-associated transport pathways in rice [32]. These findings illustrate that Cd–nutrient relationships are not fixed but depend on the physiological and chemical context.

The pattern observed in *S. variegata* differs from the Fe–Cd antagonism reported under direct Fe supplementation. Compared with Cd alone, G3Cd had higher root concentrations of both Fe and Cd; therefore, the elevated root Fe concentration does not indicate competitive exclusion of Cd uptake. Because Fe, Zn, Mn, and Cd may share components of uptake and intracellular transport networks, changes in one element can coincide with changes in others without establishing a direct causal relationship [4,31,32]. Unlike direct Fe supply, exogenous GSH introduced a distinct thiol and redox context and may have influenced multiple metal-handling processes. The concurrent increases in root Fe and Cd concentrations should therefore be interpreted as a context-dependent response rather than evidence of Fe-mediated Cd exclusion.

Because biomass contributes to tissue Fe pools, the decrease in shoot Fe concentration did not result in a significant change in shoot Fe accumulation. In contrast, the combined increases in root Fe concentration and root biomass resulted in greater root and total Fe accumulation. The larger total Fe pool is consistent with greater net whole-plant Fe acquisition and/or retention during the 10 d treatment; however, endpoint root and shoot pools do not measure transport flux. Therefore, the present data cannot determine whether root-to-shoot Fe translocation increased or decreased.

Fe is essential for photosynthetic electron transport, chlorophyll-related metabolism, and numerous redox enzymes, whereas Zn and Mn also serve structural and catalytic functions in plant metabolism [4,17]. Nevertheless, the present data do not establish that increased Fe accumulation caused the improved physiological state of G3Cd. The observed Fe pattern should therefore be interpreted as a prominent component accompanying the GSH-associated response rather than as a demonstrated mechanism of Cd tolerance. Determining whether Fe redistribution contributes directly to the GSH response will require measurements of Fe speciation, subcellular localization, transporter expression, and experimental manipulation of Fe availability.

These interpretations remain bounded by the measurement scope of the present study. Only total tissue concentrations of Cd, Fe, Zn, and Mn were determined; their subcellular distribution and chemical forms were not measured. GSH/GSSG and AsA/DHA redox couples, phytochelatins, and metal-transporter expression were also not quantified. Accordingly, the present results cannot distinguish among enhanced root sequestration, altered chelation, changes in membrane transport, cell-wall binding, or other mechanisms that could permit greater Cd accumulation with lower physiological injury. The 10 d hydroponic treatment also limits direct extrapolation to long-term field conditions. These mechanisms therefore remain targets for future validation rather than conclusions of the present study. Because GSH contains Cd-binding thiol groups, exogenous GSH may also have altered Cd speciation or availability in the nutrient solution before root uptake. The present design cannot separate this solution-phase chemical effect from GSH-induced physiological responses within the plant.

## 4. Materials and Methods

### 4.1. Plant Material, Growth Conditions, and Experimental Design

*Salix variegata* Franch. plant material was obtained from a germplasm resource area along the Jialing River in Beibei District, Chongqing, China (106.42° E, 29.85° N). Uniform rooted cuttings were hydroponically cultivated for 60 d in a controlled growth chamber before treatment. Three seedlings were maintained in each 0.7 L plastic container containing half-strength Hoagland nutrient solution [33]. Three independent containers were assigned to each treatment. The growth conditions were 25 °C during the light period and 20 °C during the dark period, with a 16 h light/8 h dark photoperiod and a light intensity of approximately 6000 lx. The nutrient solution was renewed every 3 d.

Each container was treated as one biological replicate. When a trait was measured on more than one seedling within a container before destructive sampling, the within-container mean was used as the replicate value. Destructive samples were kept container-specific and were not combined across containers. One seedling per container was retained intact until harvest for biomass and elemental analyses, while the remaining seedlings supplied tissues for destructive physiological and biochemical assays. Thus, all statistical analyses used three independent container-level biological replicates per treatment.

After 60 d of hydroponic cultivation, seedlings were treated for 10 d with CdCl_2_ at 0 or 100 μM in combination with GSH at 0, 10, 50, 250, 750, or 1500 μM. reduced glutathione (GSH; catalog no. G10019-25G; Puxitang, Beijing Jinming Biotechnology Co., Ltd., Beijing, China) and Cd were supplied simultaneously. The 12 treatments were designated as CK (0 μM GSH + 0 μM Cd), Cd (0 μM GSH + 100 μM Cd), G1–G5 (10, 50, 250, 750, and 1500 μM GSH without Cd, respectively), and G1Cd–G5Cd (the corresponding GSH concentrations in the presence of 100 μM Cd). Whenever the Hoagland nutrient solution was renewed, GSH and Cd were replenished at the designated concentration for each treatment.

Growth, morphological, and photosynthetic pigment measurements were performed for all 12 treatments. Physiological and biochemical assays were conducted for CK, Cd, G2, G3, G1Cd, G2Cd, G3Cd, and G4Cd. At harvest, G5Cd plants showed poor tissue integrity, preventing consistent collection of intact root and leaf samples for the complete physiological assay panel across all three independent containers; this treatment was therefore not included in the physiological assays. Elemental analysis was performed for CK, G3, Cd, and G3Cd. The common CK/G3/Cd/G3Cd subset provided the shared 2 × 2 factorial framework for cross-endpoint integration, whereas the additional GSH + Cd physiological treatments were retained only as supporting dose-context comparisons. The 250 μM GSH level was selected for this integration because it produced the clearest preservation of chlorophyll under Cd stress without the broad growth and pigment inhibition observed at 1500 μM; it was not assumed to be optimal for every trait.

### 4.2. Growth, Morphological Measurements, and Sample Preparation

Plant height, root length, root volume, root surface area, leaf length, leaf width, leaf area, shoot biomass, and root biomass were determined after the 10 d treatment period.

At harvest, roots were immersed in 20 mM EDTA solution for 15 min to remove metal ions adsorbed onto the root surface [34] and were then rinsed three times with distilled water. Roots were scanned using an STD1600 root scanner(Regent Instruments Inc., Québec, QC, Canada) equipped with an Epson Expression 1600 scanner (Seiko Epson Corporation, Suwa, Nagano, Japan), and root length, root volume, and root surface area were quantified using WinRHIZO software, version 4.1b (Regent Instruments Inc., Québec, QC, Canada).

For leaf morphological measurements, the fifth leaf counted downward from the shoot apex was collected. Leaves were rinsed three times with distilled water, gently blotted to remove surface moisture, and photographed. Leaf area was determined from the digital images using ImageJ [35].

The seedling retained for biomass and elemental analysis in each container was separated into roots and shoots at harvest, with stems and leaves combined as the shoot fraction. Root and shoot samples were dried to constant weight and weighed separately. The same dried material was subsequently ground and used for elemental analysis as described in Section 4.5.

### 4.3. Determination of Photosynthetic Pigments

Photosynthetic pigments were determined using an acetone–ethanol extraction procedure modified from Dang et al. [36]. Fresh leaf tissue (0.2 g) was cut into small pieces and extracted with 4 mL acetone:ethanol (1:1, *v*/*v*). Samples were sealed and maintained in darkness for 48 h. After thorough mixing, 1 mL of the extract was diluted fivefold, and absorbance was measured at 663, 645, and 470 nm using a UV–visible spectrophotometer (Shimadzu Corporation, Kyoto, Japan) spectrophotometer.

Chlorophyll a (Chl a), chlorophyll b (Chl b), and carotenoid (Car) contents were calculated according to the spectrophotometric equations described by Dang et al. [36]. Total chlorophyll (Chl t) was calculated as the sum of Chl a and Chl b, and the Chl a/b ratio was calculated as Chl a divided by Chl b. Chl a, Chl b, Car, and Chl t were expressed as mg g^−1^ fresh weight (FW), whereas Chl a/b was dimensionless.

### 4.4. Determination of Physiological and Biochemical Parameters

Root and leaf tissues were collected separately after the 10 d treatment, immediately frozen at −80 °C, and stored until analysis. For the ELISA-based assays, approximately 0.10 g of fresh tissue was homogenized on ice with extraction medium at a tissue-to-medium ratio of 1:9 (*w*/*v*) to obtain a 10% homogenate, and clarified supernatants were used for analysis. APX, POD, GR, SOD, and CAT activities and Vc, reduced GSH, H_2_O_2_, proline, and MDA contents were determined using commercial plant-specific ELISA kits (Chongqing Bonoheng Biotechnology Co., Ltd., Chongqing, China) according to the manufacturer’s instructions. The assays used analyte-specific antibody-coated microplates, HRP-labeled detection antibodies, and TMB color development; absorbance was measured at 450 nm and values were calculated from analyte-specific calibration curves. For each ELISA measurement, 10 μL of sample was mixed with 40 μL of sample diluent; values obtained from the analyte-specific calibration curve were therefore multiplied by 5. Catalogue numbers were BNH-200115P (APX), BNH-150218P (POD), BNH-170509P (GR), BNH-150205P (SOD), BNH-150206P (CAT), BNH-180029P (Vc), BNH-170526P (GSH), BNH-150268P (H_2_O_2_), BNH-150212P (proline), and BNH-150211P (MDA).

Soluble protein was determined using the bicinchoninic acid (BCA) method after ice-cold extraction and centrifugation, with absorbance measured at 562 nm. Soluble sugar was determined using the anthrone colorimetric method after hot-water extraction, with absorbance measured at 620 nm. Because approximately 0.10 g of tissue was represented by approximately 1 mL of 10% homogenate, dilution-corrected values were divided by the initial tissue fresh mass to express results per gram FW. APX, POD, and GR calibration outputs in U L^−1^ were divided by 1000 to convert them to U mL^−1^ before fresh-weight normalization, whereas SOD and CAT calibration outputs were obtained in U mL^−1^. The corresponding L-to-mL conversion was also applied to H_2_O_2_ values obtained in μmol L^−1^. APX, POD, GR, SOD, and CAT activities were expressed as U g^−1^ FW. Vc was expressed as μg g^−1^ FW, GSH and proline as ng g^−1^ FW, H_2_O_2_ as μmol g^−1^ FW, MDA as nmol g^−1^ FW, and soluble sugar and soluble protein as mg g^−1^ FW.

### 4.5. Determination of Cd, Fe, Zn, and Mn Concentrations and Accumulation

The dried root and shoot samples obtained as described in Section 4.2 were ground before digestion. An accurately weighed portion (approximately 0.1000–0.3000 g) of each dried, ground sample was transferred into a clean and dry polytetrafluoroethylene digestion vessel. Ten milliliters of high-purity concentrated HNO_3_ was added, and the sample was mixed for 1 min. The vessels were then heated at 120 °C for 20 min for pre-digestion.

After cooling, an additional 5 mL of high-purity concentrated HNO_3_ was added. The vessels were sealed and subjected to microwave digestion using a Touchwin 2.0 microwave digestion system following the programmed digestion cycle used by the analytical laboratory. A comparable HNO_3_ microwave digestion procedure using this digestion platform has been reported for elemental determination in plant tissues [37].

After microwave digestion, the vessels were cooled to room temperature and opened carefully. The digests were heated at 180 °C for 30 min to remove excess acid until approximately 1 mL of clear solution remained. Each digest was quantitatively transferred into a 50 mL centrifuge tube using ultrapure water in three rinses and brought to a final volume of 50 mL. The solutions were mixed for 1 min and filtered through a 0.22-μm membrane. Appropriate dilutions were prepared when necessary.

Cd, Fe, Zn, and Mn concentrations were determined using a NexION 300D inductively coupled plasma mass spectrometer (ICP-MS; PerkinElmer,, PerkinElmer, Inc., Shelton, CT, USA). The instrument used high-purity Ar (≥99.9999%) and element-specific Standard and KED modes. For KED measurements, the Cell Gas A setting was 0.8 mL min^−1^. The auxiliary and plasma gas flow rates were 1.2 and 17 L min^−1^, respectively; the instrument cooling system was maintained at 20 °C, and the acquisition time was 2–3 min. Quantification was performed using external calibration with internal-standard correction. External calibration standards were prepared from a certified 32-element mixed standard solution (GSB 04-4306-2025; 100 mg L^−1^ per element) [38], which included Cd, Fe, Mn, and Zn. A five-element ICP-MS internal-standard solution (BWB2604-2016; Bi, Ge, In, Rh, and Re; 100 mg L^−1^ each) was used for internal-standard correction [39]. Calibration curves were fitted through zero; the analytical-run correlation coefficients were 0.999809 for Cd, 0.997694 for Fe, 0.999823 for Zn, and 0.997660 for Mn. Background-corrected readings for the analytical blank were negative for all four elements and were reported as not detected. A laboratory quality-control solution with a nominal concentration of 100 μg L^−1^ was analyzed in triplicate; the mean recoveries were 100.54%, 104.27%, 103.22%, and 101.42% for Cd, Fe, Zn, and Mn, respectively (Table S12). These values represent solution-level quality control. No plant-matrix certified reference material or sample matrix-spike recovery test was included in this analytical batch. Element concentrations were expressed as μg g^−1^ dry weight (DW).

Element accumulation was calculated separately for each biological replicate by matching the elemental concentration with the corresponding root or shoot biomass from the same biological replicate:

Shoot accumulation = shoot concentration × shoot biomass

Root accumulation = root concentration × root biomass

Total accumulation = shoot accumulation + root accumulation

Element accumulation was expressed as μg. Root allocation was calculated as:

Root allocation (%) = root accumulation/total accumulation × 100

### 4.6. Statistical Analysis

All statistical analyses were performed using R version 4.5.1 (R Core Team, Vienna, Austria). The container was the experimental unit, and data are presented as means ± standard deviation (SD) of three independent container-level biological replicates. GSH concentration was treated as a categorical factor in all factorial analyses.

Growth and morphological variables were analyzed using a 2 × 6 factorial design comprising two Cd levels (0 and 100 μM) and six GSH levels (0, 10, 50, 250, 750, and 1500 μM). Type III analysis of variance (ANOVA) was used to test the main effects of Cd and GSH and their interaction. Within each Cd background, each non-zero GSH treatment was compared with the corresponding 0 μM GSH treatment using Dunnett-adjusted comparisons [40]. The same 2 × 6 factorial procedure was applied to the photosynthetic pigment variables.

For physiological and biochemical variables, CK, G3, Cd, and G3Cd provided the common 2 × 2 factorial structure shared with the elemental dataset, with Cd at 0 or 100 μM and GSH at 0 or 250 μM. Root and leaf data were analyzed separately using Type III ANOVA. Partial eta-squared (ηp^2^) was calculated for the Cd × GSH interaction. Planned GSH simple effects were evaluated as G3 versus CK and G3Cd versus Cd, and the two simple-effect *p* values within each tissue and variable were adjusted using the Holm procedure. The additional Cd-stressed treatments G1Cd, G2Cd, G3Cd, and G4Cd were compared with the Cd-only treatment using Dunnett-adjusted comparisons to provide supporting GSH dose context under Cd stress.

Element concentrations in CK, G3, Cd, and G3Cd were analyzed as a 2 × 2 factorial design. Root and shoot data were analyzed separately using Type III ANOVA, and ηp^2^ was calculated for Cd × GSH interactions. GSH simple effects were evaluated within the Cd-free and Cd-stressed backgrounds. *p* values for the planned element comparisons were adjusted using the Benjamini–Hochberg false-discovery rate (BH-FDR) procedure [41].

For element accumulation and root-allocation variables, G3Cd was compared with the Cd-only treatment, and the resulting *p* values were corrected using the BH-FDR procedure. Residual normality and homogeneity of variance were evaluated using the Shapiro–Wilk test [42] and Levene’s test [43], respectively. Statistical significance was accepted at *p* < 0.05, adjusted *p* < 0.05, or FDR < 0.05, as appropriate.

## 5. Conclusions

Across a broad GSH concentration series, *S. variegata* did not show a single monotonic protective response to Cd. Low GSH concentrations improved selected root traits, 250 μM GSH produced the clearest chlorophyll preservation among the tested Cd treatments, and 1500 μM GSH inhibited several growth and pigment traits. At 250 μM GSH, H_2_O_2_ and MDA decreased in both roots and leaves, while several antioxidant and osmotic variables showed tissue-dependent responses. This physiological adjustment coincided with a 165% increase in root Cd accumulation and a 117% increase in total Cd accumulation, demonstrating that lower shoot Cd concentration did not represent a lower whole-plant Cd burden. Fe homeostasis was also strongly altered, particularly in roots. These results support interpreting exogenous GSH responses as dose- and tissue-dependent physiological adjustment rather than simple Cd exclusion or uniform antioxidant activation. The cellular mechanisms that permit improved physiological status under greater internal Cd burden remain to be resolved. Because these conclusions derive from a 10 d hydroponic experiment, their persistence and practical relevance require validation in longer-term soil and field studies.

## Data Availability

The data supporting the findings of this study are provided within the article and its Supplementary Materials. Additional data are available from the corresponding author upon reasonable request.

## Supplementary Materials

The following supporting information can be downloaded at https://www.mdpi.com/article/10.3390/plants15182801/s1, Figure S1: Complete growth and morphological responses of *Salix variegata* across the GSH dose series under control and Cd-stress conditions; Figure S2: Root physiological responses in the 250 μM GSH-centered 2 × 2 core design; Figure S3: Leaf physiological responses in the 250 μM GSH-centered 2 × 2 core design; Figure S4: Supporting GSH dose context for root and leaf physiological responses under Cd stress; Figure S5: Effects of 250 μM GSH on Cd and essential-element concentrations in roots and shoots; Figure S6: Effects of 250 μM GSH on shoot, root, and total accumulation of Cd, Fe, Zn, and Mn; Table S1: Experimental structure and statistical framework; Table S2: Type III factorial ANOVA for growth and morphological traits; Table S3: Significant Dunnett-adjusted GSH contrasts for growth and morphology; Table S4: Type III factorial ANOVA for photosynthetic pigments; Table S5: Significant Dunnett-adjusted GSH contrasts for photosynthetic pigments; Table S6: Type III factorial ANOVA for root and leaf physiological variables in the 2 × 2 core design; Table S7: Planned GSH simple effects for root and leaf physiological variables in the 2 × 2 core design; Table S8: Dunnett-adjusted physiological responses to GSH dose under Cd stress; Table S9: Type III factorial ANOVA for Cd, Fe, Zn, and Mn concentrations; Table S10: GSH simple effects on Cd, Fe, Zn, and Mn concentrations; Table S11: Effects of 250 μM GSH on element accumulation and root allocation under Cd stress; Table S12: Analytical blank and solution-level quality-control results for ICP-MS analysis.
