# Supplementary material for: Dose-Dependent Glutathione Modulation of Physiological Responses and Metal Accumulation in Salix variegata Under Cadmium Stress
Source: Plants (Basel). 2026 Sep 12;15(18):2801. doi: 10.3390/plants15182801 (PMC13610906; doi:10.3390/plants15182801)
Supplement: Supplementary file 1 [file plants-15-02801-s001.zip › plants-4542082_Supplementary_Figures.pdf]

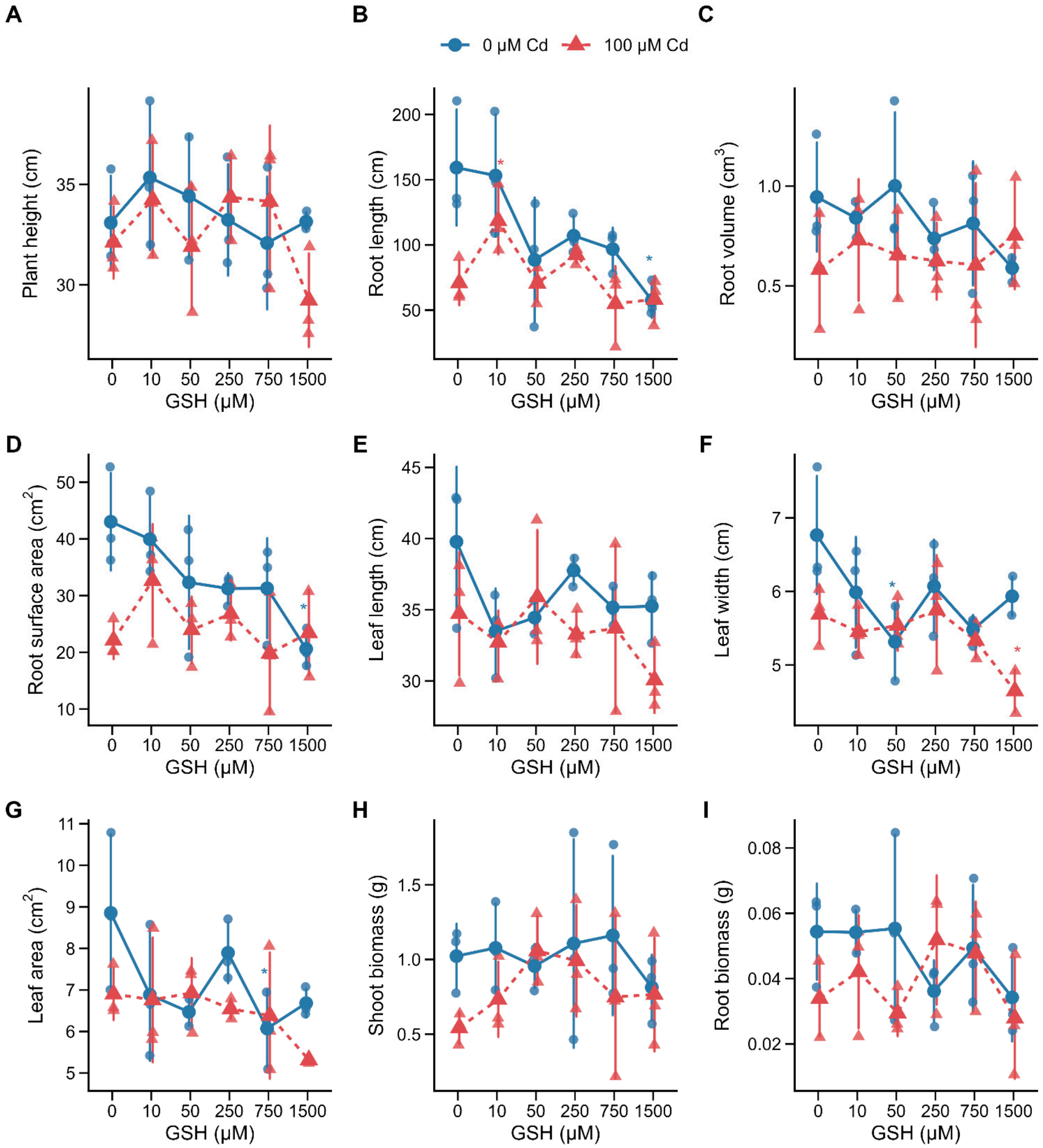

Figure S1. Complete growth and morphological responses of Salix variegata across the GSH dose series under control and Cd-stress conditions. Plant height (A), root length (B), root volume (C), root surface area (D), leaf length (E), leaf width (F), leaf area (G), shoot biomass (H), and root biomass (I) were measured in plants supplied with 0, 10, 50, 250, 750, or 1500 μM GSH under 0 or 100 μM Cd. Small symbols represent individual biological replicates, and large symbols with error bars represent means ± SD (n = 3). Blue asterisks indicate Dunnett-adjusted comparisons with CK within the Cd-free background, whereas red asterisks indicate comparisons with the Cd-only treatment within the Cd background. *P_adj < 0.05, **P_adj < 0.01, and ***P_adj < 0.001.

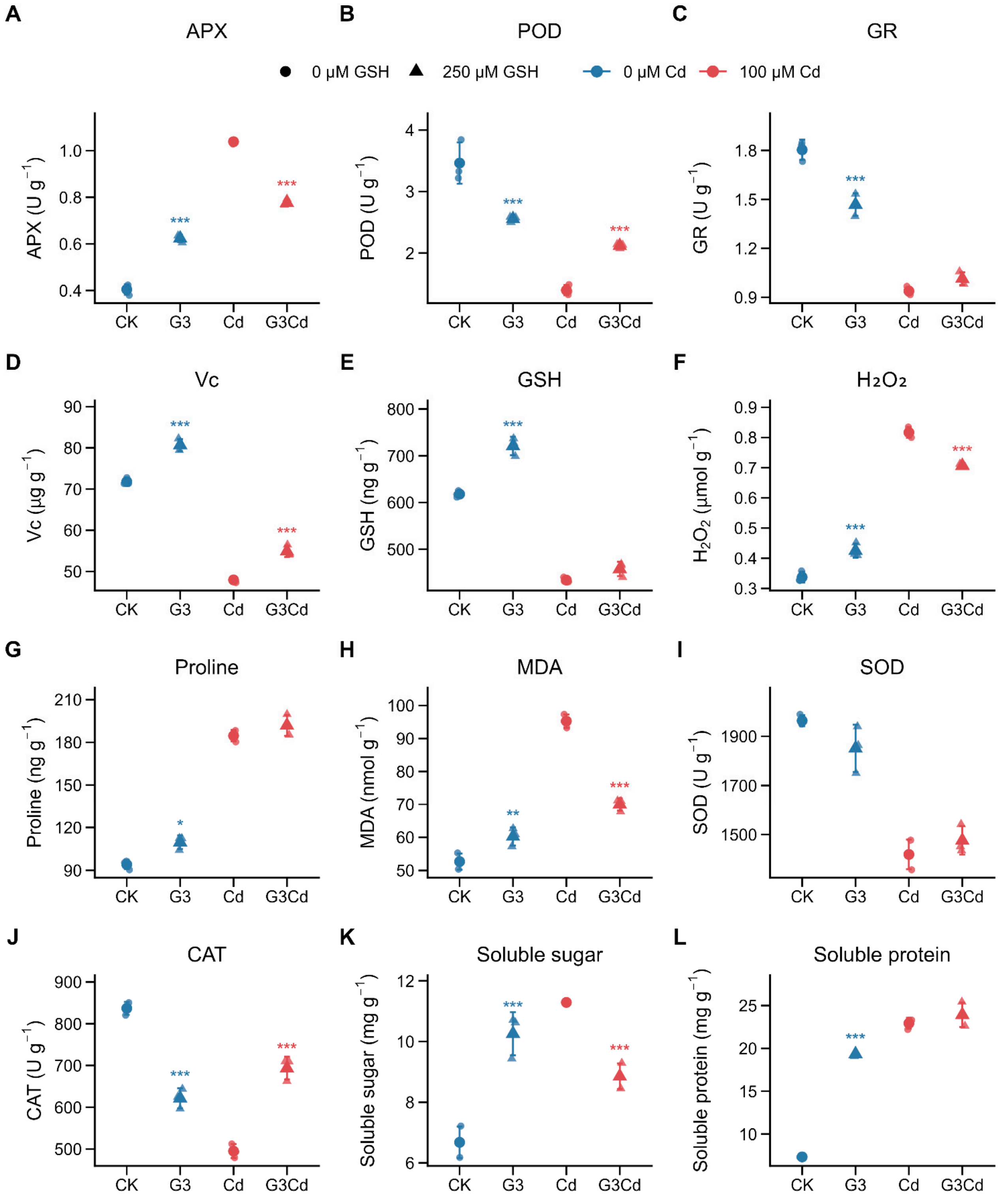

Figure S2. Root physiological responses in the 250 µM GSH-centered 2 × 2 core design. APX (A), POD (B), GR (C), Vc (D), GSH (E), H_2O_2 (F), proline (G), MDA (H), SOD (I), CAT (J), soluble sugar (K), and soluble protein (L) are shown for CK, G3, Cd, and G3Cd roots. Blue and red symbols denote 0 and 100 µM Cd, respectively; circles and triangles denote 0 and 250 µM GSH, respectively. Small symbols represent individual biological replicates, and large symbols with error bars represent means ± SD (n = 3). Asterisks above G3 and G3Cd indicate the planned GSH simple effects G3 vs CK and G3Cd vs Cd, respectively, using Holm-adjusted P values. *P_adj < 0.05, **P_adj < 0.01, and ***P_adj < 0.001.

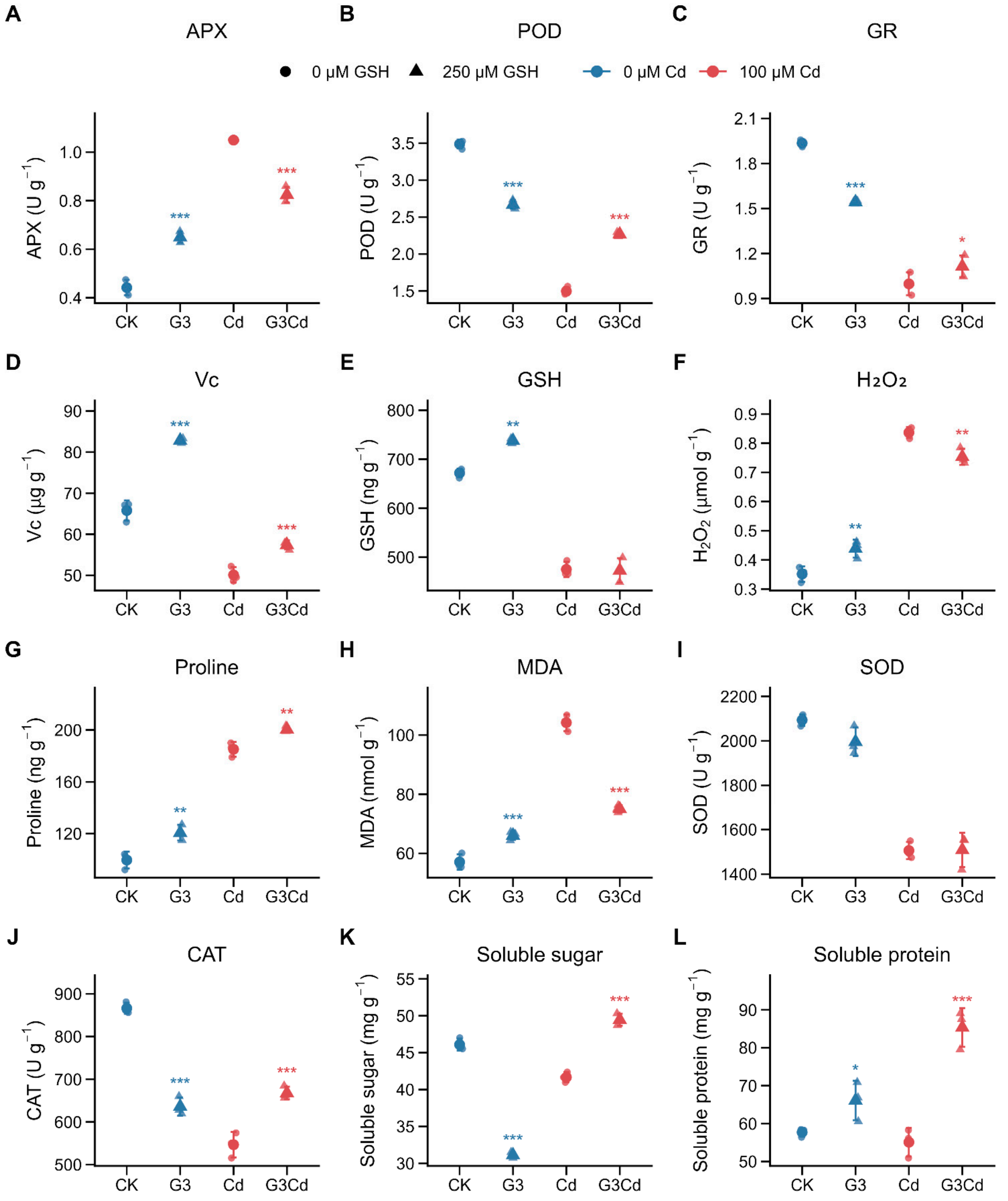

Figure S3. Leaf physiological responses in the 250 μM GSH-centered 2 × 2 core design. APX (A), POD (B), GR (C), Vc (D), GSH (E), H_2O_2 (F), proline (G), MDA (H), SOD (I), CAT (J), soluble sugar (K), and soluble protein (L) are shown for CK, G3, Cd, and G3Cd leaves. Blue and red symbols denote 0 and 100 μM Cd, respectively; circles and triangles denote 0 and 250 μM GSH, respectively. Small symbols represent individual biological replicates, and large symbols with error bars represent means ± SD (n = 3). Asterisks above G3 and G3Cd indicate the planned GSH simple effects G3 vs CK and G3Cd vs Cd, respectively, using Holm-adjusted P values. *P_adj < 0.05, **P_adj < 0.01, and ***P_adj < 0.001.

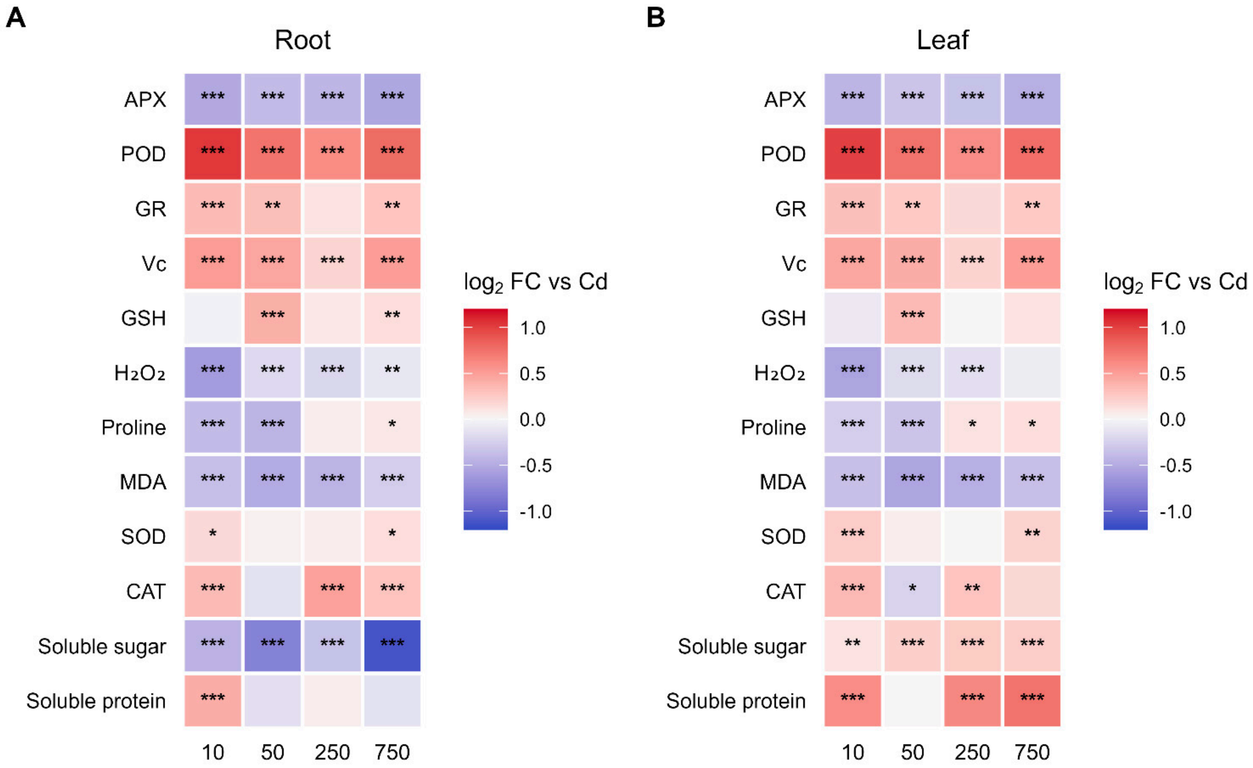

Figure S4. Supporting GSH dose context for root and leaf physiological responses under Cd stress. Heatmaps show $\log_2$ fold changes relative to the Cd-only treatment for roots (A) and leaves (B) supplied with 10, 50, 250, or 750 μM GSH in the presence of 100 μM Cd. The variables are APX, POD, GR, Vc, GSH, H_2O_2, proline, MDA, SOD, CAT, soluble sugar, and soluble protein. Asterisks indicate Dunnett-adjusted comparisons of each GSH + Cd treatment with the Cd-only treatment within the same tissue and variable. These comparisons provide supporting dose-response context and are not part of the 2 × 2 factorial interaction model. *P_adj < 0.05, **P_adj < 0.01, and ***P_adj < 0.001.

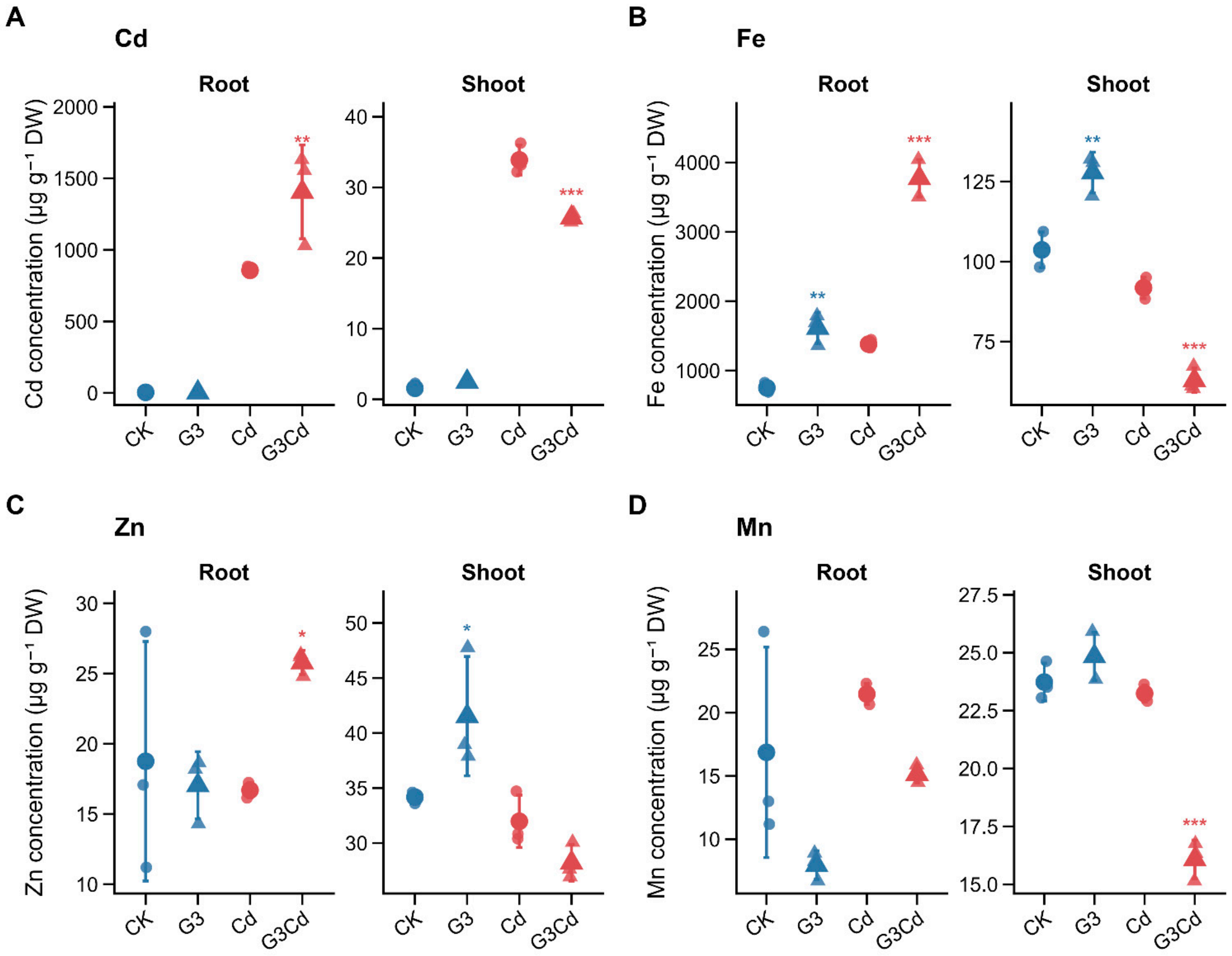

Figure S5. Effects of 250 μM GSH on Cd and essential-element concentrations in roots and shoots. Cd (A), Fe (B), Zn (C), and Mn (D) concentrations are shown for CK, G3, Cd, and G3Cd plants. Element concentrations are expressed as μg g^{-1} DW. G3 and G3Cd denote 250 μM GSH in the absence and presence of 100 μM Cd, respectively. Small symbols represent individual biological replicates, and large symbols with error bars represent means ± SD (n = 3). Blue asterisks indicate BH-FDR-adjusted comparisons between G3 and CK, whereas red asterisks indicate comparisons between G3Cd and Cd. *FDR < 0.05, **FDR < 0.01, and ***FDR < 0.001.

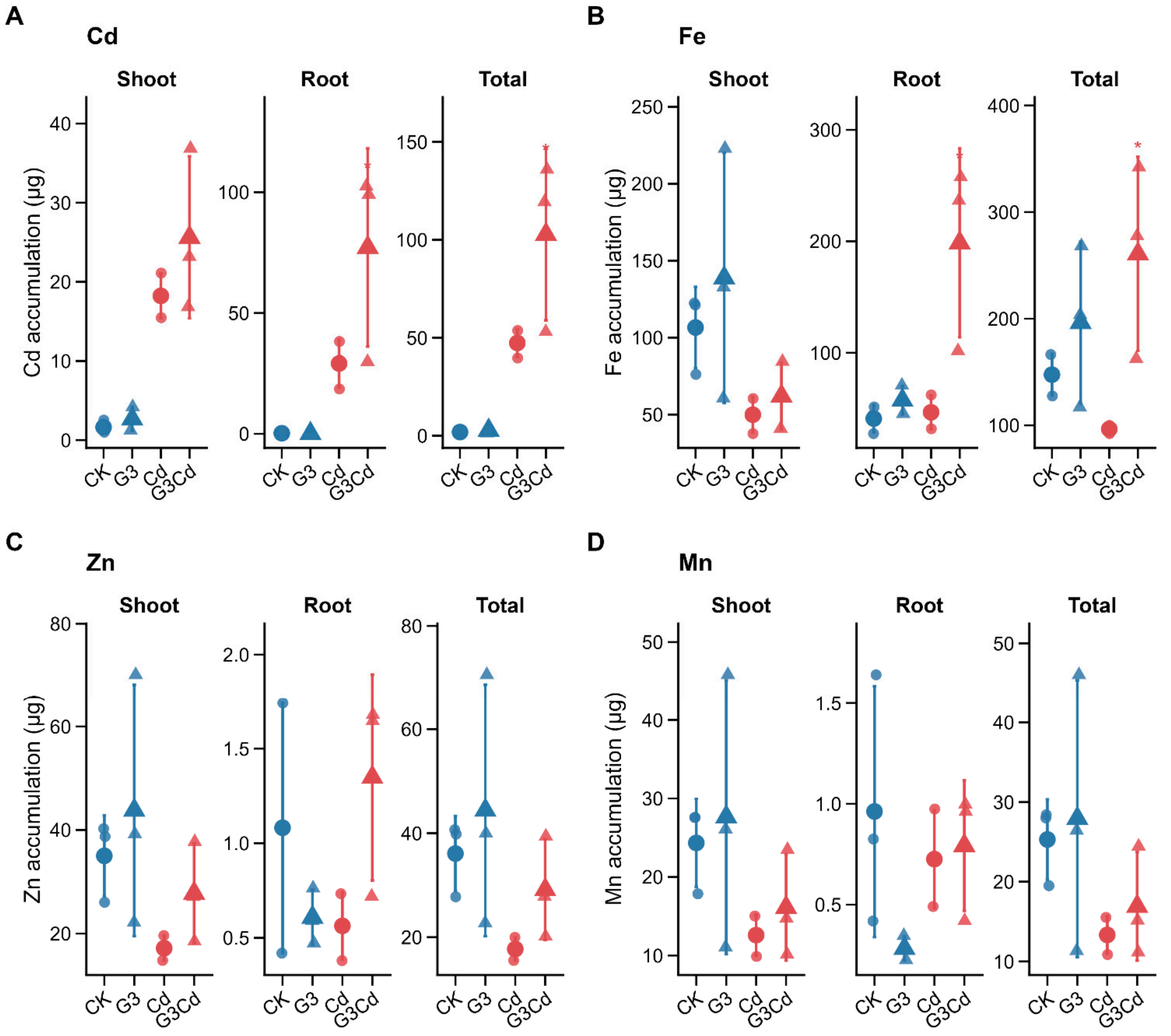

Figure S6. Effects of 250 μM GSH on shoot, root, and total accumulation of Cd, Fe, Zn, and Mn. Shoot, root, and total accumulation of Cd (A), Fe (B), Zn (C), and Mn (D) are shown for CK, G3, Cd, and G3Cd plants. Element accumulation was calculated from tissue concentration and the corresponding biomass of the same biological replicate and is expressed in μg. Small symbols represent individual biological replicates, and large symbols with error bars represent means ± SD (n = 3). Red asterisks indicate BH-FDR-adjusted comparisons between G3Cd and Cd. *FDR < 0.05, **FDR < 0.01, and ***FDR < 0.001.
